# The Doctor

**Published:** 1916-02

**Authors:** 


### EDITORIAL DEPARTMENT

MRS. F. E. DANIEL, Publisher and Managing Editor.
DR. R. H. L. BIBB, Associate Editor.

CONTRIBUTING EDITORS:

Dr. H. Sheridan Baketel, New York, editor *Medical Times;* Dr. G. Henri Bogart, Paris, Ill.; Dr. M. M. Carrick, Dallas, Texas; Dr. Edward H. Carey, Dean Medical Department, Baylor University, Dallas, Texas; Dr. W. L. Crosthwaite, Waco, Texas; Dr. T. D. Crothers, Hartford, Conn.; Dr. Oscar Dowling, New Orleans, President Louisiana State Board of Health; Havelock Ellis, M. D., London, Eng.; Dr. May Agnes Hopkins, Dallas, Texas; Dr. A. W. Fly, Galveston, Texas; Dr. G. B. Foscue, Waco, Texas; Dr. E. S. Goodhue, Holualoa, Kona, Hawaii; Dr. Joe Gilbert, Austin, Texas; Dr. Charles H. Hughes, St. Louis, editor *Alienist and Neurologist;* Dr. George H. Lee, Galveston, Texas; Dr. John T. Moore, Houston, Texas; Dr. Frank Paschal, San Antonio, Texas; Dr. John Preston, Austin, Texas, Superintendent State Lunatic Asylum; Dr. G. Wilse Robinson, Kansas City, Mo.; Dr. Bacon Saunders, Fort Worth, Texas; Dr. Allen J. Smith, Philadelphia, Pa., Medical Department, University of Pennsylvania; Dr. Z. T. Scott, Austin, Texas; Dr. Walter S. Sutton, Kansas City, Mo.; Dr. A. E. Sweatland, Nacogdoches, Texas; Dr. John S. Turner, Dallas, Texas; Dr. Charles Venable, San Antonio, Texas.

---

### The Doctor.

The doctor he comes and he holds my weary hand,
And he says I'll soon be better, and that soon he'll let me stand;
He promises the roses to my cheeks shall come again,
And he laughs away the fever, and he jokes away the pain.
Through the long, long night I suffer; weird the dreams that
come to me.
Quaint the thoughts that I am thinking; strange the sights I
can see.
But the sunbeams of the morning bring the doctor up the stairs
And the heart of me is lighted of a thousand different cares.
There is courage in the twinkle of his kindly smiling eyes.
And before his merry laughter fly a thousand fears and sighs;
And the thoughts that have been dreary change to pleasant ones
and gay.
When the good old kindly doctor smiles the doubts and dreads
away.
For the doctor he comes singing and he sits beside my bed
And he lifts my weary spirits, as the pillow lifts my head;
And the fever seems to leave me and the pains are not severe
And I'm better for his presence and I'm stronger for his cheer.
The doctor he is clever, sure and certain to his skill,
And his people long have praised him for his work among the ill;
But it's not his wisdom only that the life of us insures
And it's not the pills and tonics, but the heart of him that cures.

*—Clipped.*

